# Culture-negative endocarditis with neurologic presentations and dramatic response to heparin: a case report

**DOI:** 10.1186/s12879-020-05206-0

**Published:** 2020-07-06

**Authors:** Hossein Sheibani, Mohammad Salari, Elham Azmoodeh, Amirhessam Kheirieh, Sara Chaghazardi

**Affiliations:** 1 Clinical Research Development Unit, Imam Hossein Hospital, Shahroud University of Medical Science, 3616911151, Imam Ave, Shahroud, Iran; 2 Student Research Committee, School of Medicine, Shahroud University of Medical Science, Shahroud, Iran; 3 Cardiologist, Tehran, Iran

**Keywords:** Bacterial endocarditis, Heparin, Echocardiography, Intracranial embolism

## Abstract

**Background:**

Blood culture-negative endocarditis (BCNE) is diagnosed in 2–7% of patients with infective endocarditis (IE) and recent antibiotic use is a known risk factor. Altered mental status may be a presenting symptom. Besides empiric antibiotics, intravenous anticoagulation using heparin may have a role in the management of such patients.

**Case presentation:**

A 23-year-old male patient was referred to our center with fever, altered mental status and abnormal gait. Neurologic examination revealed Wernicke’s aphasia. Cardiac auscultation revealed systolic murmur at the left sternal border. ECG (electrocardiogram) was unremarkable. Brain MRI showed multiple cerebellar lesions. Transthoracic echocardiography (TTE) demonstrated three large masses on the right ventricle (RV), tricuspid valve (TV), and anterior mitral valve (MV) leaflet. Blood cultures (three sets) were negative. Intravenous heparin therapy was administered. After 48 h, the second TTE demonstrated that one valvular lesion disappeared and the other two lesions showed a significant decrease in size. The patient’s neurological symptoms resolved gradually. Further workup for collagen vascular disorders did not show any abnormality.

**Conclusion:**

BCNE should be considered in patients with fever and neurologic manifestations. TTE should be performed to detect valvular abnormalities. Intravenous heparin could be used in such patients when TTE demonstrate valvular vegetations.

## Background

Blood culture-negative endocarditis (BCNE) is endocarditis in which blood cultures using usual laboratory methods remain sterile. This condition has a wide incidence rate (35 to 70% of all cause of endocarditis) depending on host characteristics and geographical area [1, 2].

BCNE is difficult to diagnosis [3] since positive blood culture which is one of the major criteria to diagnose infective endocarditis (IE) is absent in 2–7% of blood samples. These patients occasionally present with signs and symptoms highly suggestive of IE. A major reason for negative blood culture is recent antibiotic use by patients which render blood culture negative [4]. Additionally, routinely used culture media may not diagnose the causative organisms including fastidious bacteria or fungi [4, 5].

Antibiotic treatment has been suggested for BCNE [6]. Guidelines have been published regarding empiric antimicrobial treatment for BCNE [7]. Combination of amoxicillin (6 weeks) and gentamicin (3 weeks) was shown to be a superior antimicrobial treatment in a previous study on patients with community-acquired BCNE [8].

We present a young patient who presented with fever, abnormal gait, and altered mental status. Echocardiographic findings, the course of the condition, and response to administered antibiotics are described. In addition, the role of intravenous heparin in the management of the patient is elucidated.

## Case presentation

A 23-year-old previously healthy man was initially visited in a local health center due to fever, nausea, vomiting and diarrhea as well as eye pain. With the presumptive diagnosis of a viral infection and keratoconjunctivitis, he was prescribed parenteral ceftriaxone (1 gam single dose), metoclopramide (10 mg orally every 8 hours) and hyoscine, an anticholinergic agent, (10 mg orally every 12 h). However, his initial symptoms including eye pain did not resolve after 1 week. Therefore, the patient presented to another healthcare center where cefixime, a third-generation cephalosporin, (400 mg orally once daily) and sulfacetamide ophthalmic drop (2 drops every 3 hours) were prescribed for him. He showed relative response to the new antibiotics and although his eye condition improved significantly, fever did not resolve. Meanwhile he developed alerted mental status and abnormal gait. At this point, the patient was referred to our center. Upon presentation, he had fever (oral temperature of 38.8 °C), non-productive coughs, altered mental status, slurred speech, and abnormal gait. There was no evidence of weight loss or night sweats. Heart rate, respiration and blood pressure were within normal range. On neurologic examination, the Romberg test was negative and the patient’s gait was wide base without dynamic apraxia. He had Wernicke aphasia and disturbed writing and reading ability. On cardiac examination, systolic murmur was detected at the left sternal border.

Past medical history was unremarkable except for febrile convulsions in his childhood for which no pharmacotherapy had been initiated. History for psychological and congenital diseases was negative. He did not consume alcohol and did not smoke cigarettes or illicit substances. He did not use any regular medications. He had a normal diet. He served in the military with a middle-class socioeconomic status. He had no previous exposure to pets and did not have recent travel history to other countries.

**Fig. 1:**
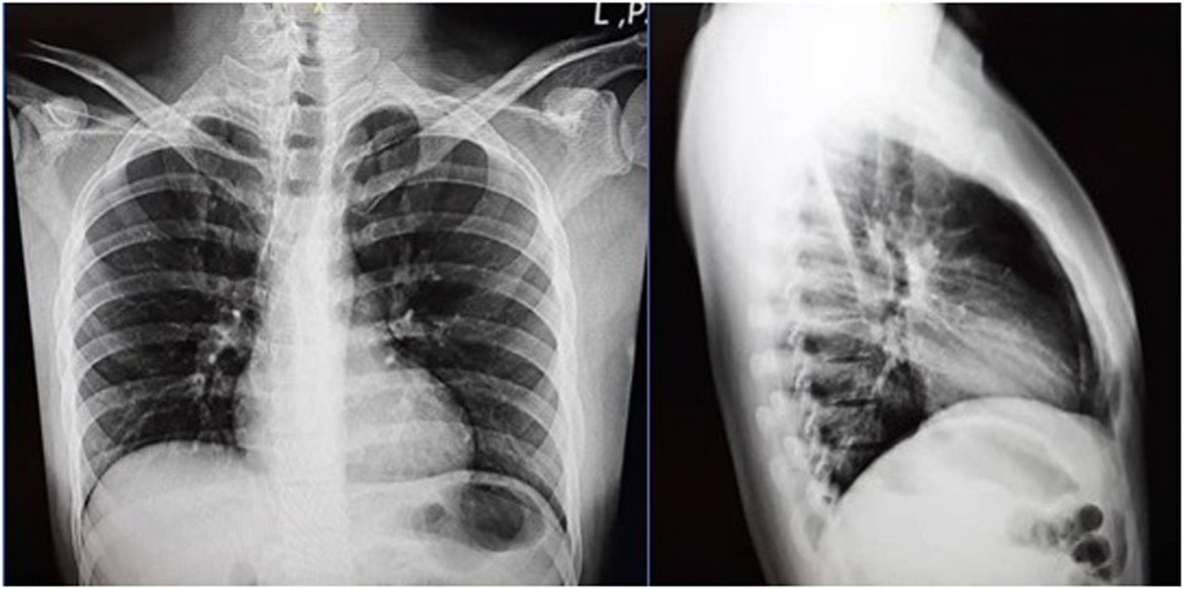
Chest radiography did not show any abnormalities

Initial laboratory tests at the emergency department (ED) showed white blood cell (WBC) count of 14,100/mm^3^ (neutrophils = 85%), haematocrit of 46%, platelet count of 156,000/mm^3^, blood sugar of 161 mg/dL, erythrocyte sedimentation rate (ESR) of 23 mm/h, c-reactive protein (CRP) of 49 mg/L, CK-MB = 28 U/L. Serum BUN (blood urea nitrogen), creatinine, and calcium level were within normal range. ECG at the ED did not show any remarkable abnormality. Chest X-Ray was done and demonstrated no remarkable points (Fig. 1). The patient was transferred to the intensive care unit (ICU) and received empiric antibiotic treatment with ceftriaxone (2 g intravenously every 12 h) and acyclovir (400 mg orally every eight hours).

**Fig. 2:**
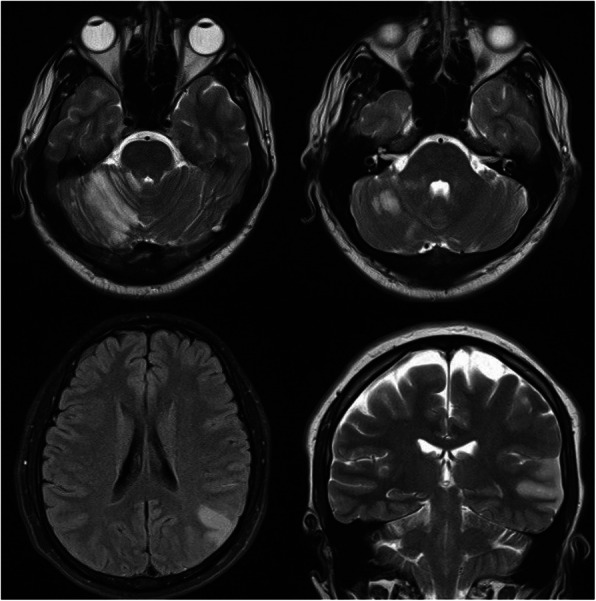
Brain MRI (magnetic resonance imaging) showing multiple cerebellar and parieto-occipital ischemic lesions

Considering abnormal neurologic findings, brain MRI was performed which demonstrated multiple cerebellar and parieto-occipital ischemic lesions (Fig. 2). High signal T2/FLAIRE/DWI areas involving the grey matter and white matter were seen at the right cerebellar hemisphere (56 × 27 mm) and left parieto-occipital (50 × 30 mm) lobes suggestive of subacute infarction. Chest, abdomen and pelvic CT scans did not show any abnormality.

**Fig. 3:**
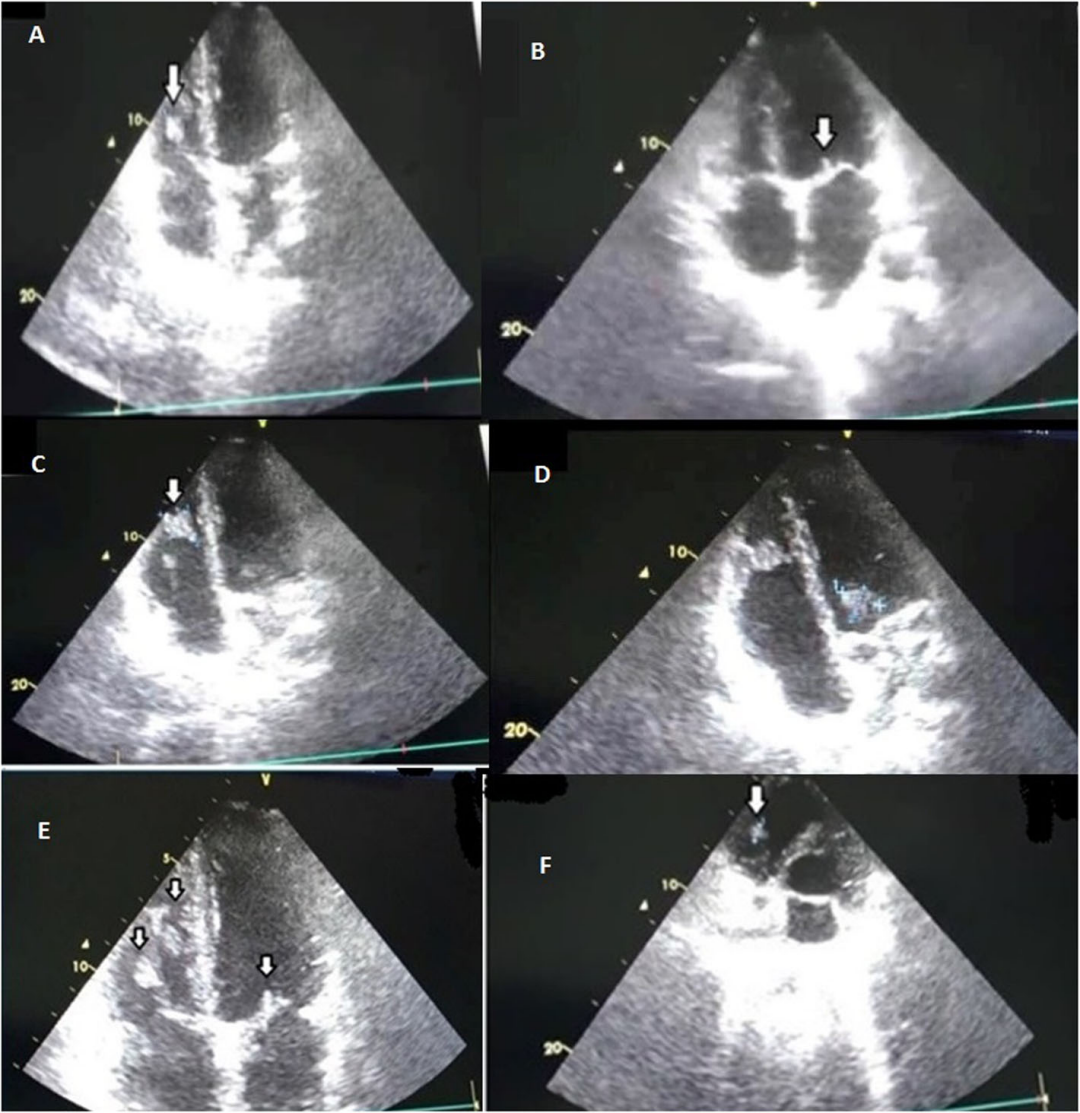
Transthoracic echocardiography (TTE): **a** four-chamber view showing a large-sized vegetation (arrow) seen on the right ventricular side of the leaflet of the tricuspid valve (TV); **b** four-chamber view showing a vegetation (arrow) on the ventricular side of the anterior leaflet of the mitral valve; **c** four-chamber view showing a large vegetation (arrow) on the lateral wall of the right ventricle; **d** Parasternal long axis view showing two large vegetations on mitral valve and lateral wall of RV; **e** Parasternal long axis view showing three large vegetations on mitral and tricuspid valves and lateral wall of RV; **f** Parasternal short-axis view showing a large vegetation on the ventricular side of the leaflet of TV

The patient’s condition did not change after 4 days. On the 4th day, platelet count decreased to 120,000/mm3. Considering the brain imaging findings and the presence of systolic murmur, transthoracic echocardiography (TTE) was performed with presumptive diagnosis of IE as the source of the cerebellar lesions. TTE revealed multiple mobile large masses on the lateral wall of the right ventricle (RV) (24 × 18 mm), on RV side of the lateral leaflet of the tricuspid valve (TV) (13 × 12 mm), and left ventricular (LV) side of the anterior leaflet of the mitral valve (MV) (10 × 8 mm) (Fig. 3). The ventricular and valvular functions were normal except mild tricuspid regurgitation (TR) with central jet. Considering TTE findings, three sets of blood samples from three different locations were obtained for blood culture. All three samples were negative for any bacterial growth. Urine sample likewise did not show any positive culture. The patient’s high temperature and leukocytosis persisted. Platelet count increased from 120,000 to 180,000/mm^3^. Other serology tests such as VDRL/RPR (rapid plasma regain) for syphilis, human immune deficiency (HIV) antibody, human T-lymphotropic virus-1 (HTLV-1) antibody, hepatitis B surface antigen/antibody, hepatitis C virus antibody, antinuclear antibody (ANA), Wright test, 2-mercaptoethanol test (2ME) for brucellosis, Widal test, anticardiolipin, and antiphospholipid were in normal range or negative.

Considering the location of the masses on the ventricular wall and MV and their relatively large size heparin (1000 units/hour) as well as vancomycin (1 g every 12 h) were initiated., About 4 h after initiating these treatments, the patient developed thrombocytopenia (platelet count of 86,000/mm3) and respiratory symptoms including productive coughs and tachypnea (respiratory rate of 19/min). However, blood oxygen saturation did not fall and no change in blood pressure was observed. At this stage, heparin was discontinued. Although the patient was receiving parenteral antibiotic, leukocytosis did not resolve (WBC = 22,600/mm^3^).

We decided to start anticoagulant treatment with heparin (800 units per hour) again after 12 h of discontinuation. Also, it was decided to add doxycycline (100 mg orally every 12 h) and meropenem (1 g intravenously every 8 h). Laboratory tests showed WBC of 18,600/mm^3^ (neutrophil = 80%), platelet of 186,000/mm3, and INR (international normalized ratio) of 1.

**Fig. 4:**
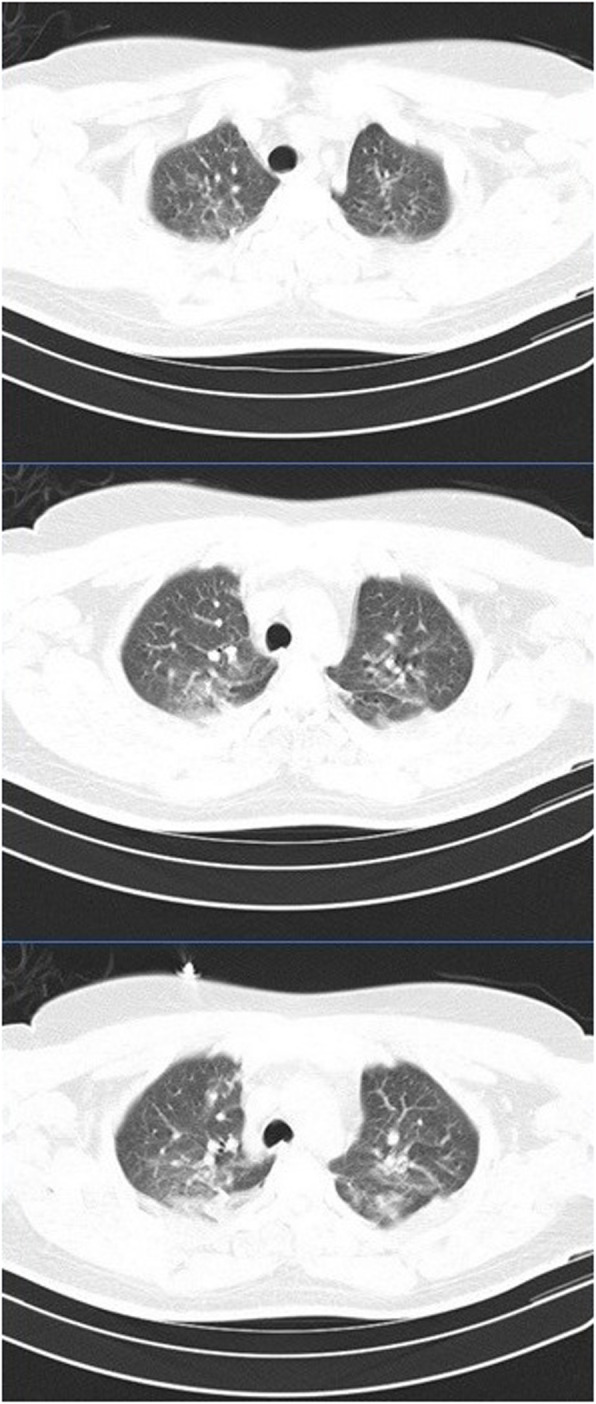
Chest CT did not reveal any lesions such as acute parenchymal injury or pulmonary embolism

On follow-up after 48 h of heparin and new antibiotics initiation, fever did not resolve. With suspicion of pulmonary embolization, TTE was repeated. It showed that the lesion on the TV disappeared completely. Also, the lesions in the RV and on MV showed significant decrease in size. Contrast-enhanced chest CT scan did not show any pulmonary lesions (Fig. 4). CT angiography was not available at that time.

On the next follow-up, the patient’s neurological and respiratory symptoms started to resolve gradually but not completely.

Heparin was discontinued on the 8th day. We decided to refer for more complete workup including collagen-vascular diseases as a potential cause of BCNE. Further transthoracic echocardiography examinations performed in the tertiary center after 1, 2 and 3 weeks did not show any vegetation, atrial septal defect or patent foramen ovale. Neurological and respiratory symptoms such as disorientation, dysarthria, ataxia, Wernicke aphasia and persistent cough resolved completely. Serologic evaluation and immunofluorescence assay for microorganisms such as *Coxiella burnetii*, *Tropheryma whipplei*, *Mycoplasma*, *Legionella*, *Bartonella* and *Borrelia* were negative. PPD test and sputum culture for *Mycobacterium* were negative. Blood culture after 14 days was negative. Evaluation for *Aspergillus* and *Candida* (anaerobic culture) was negative. Anti-heparin antibodies were not detected.

**Fig. 5:**
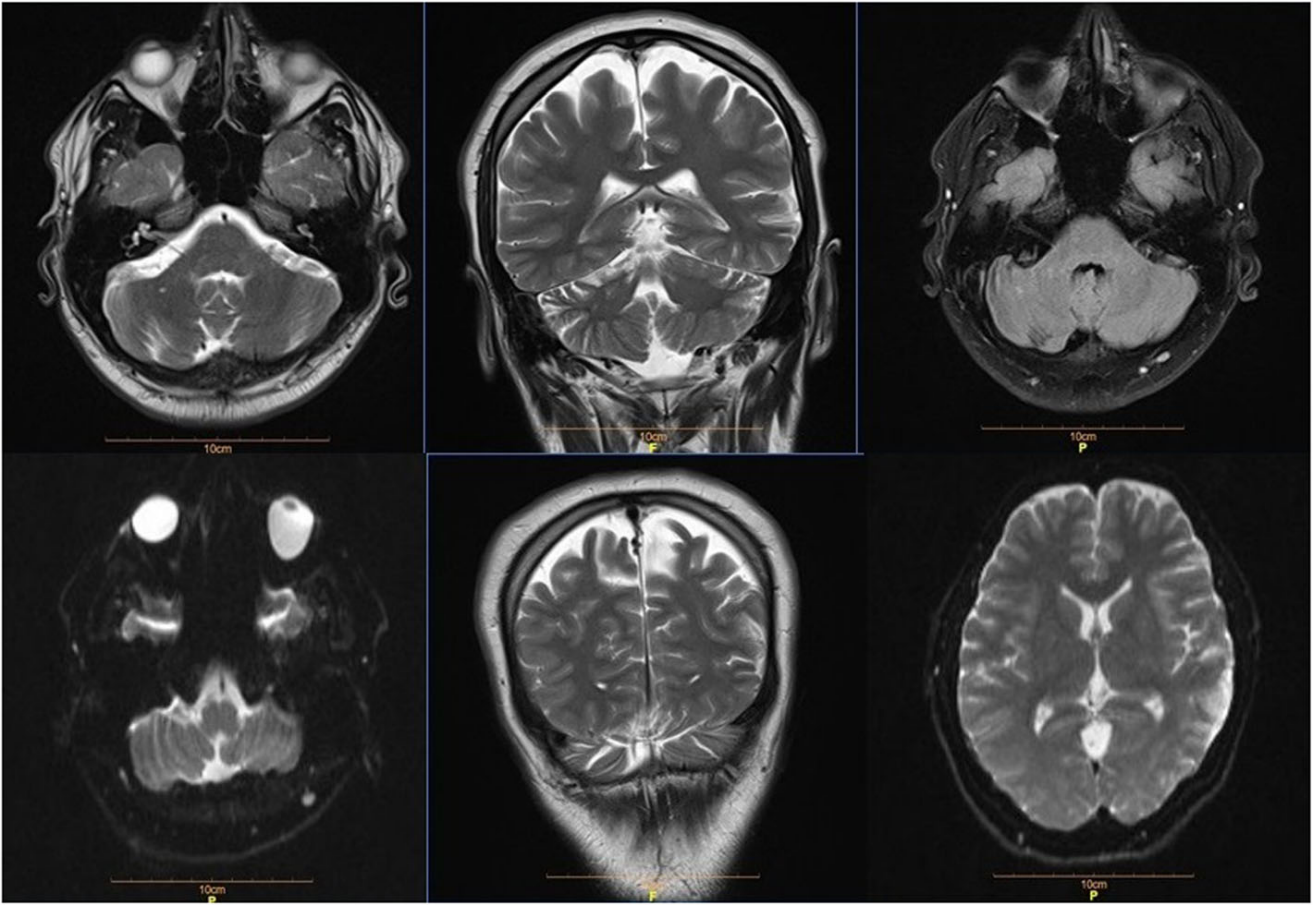
Brain MRI was done 3 months later that demonstrated relative improvement in prior ischemic lesions without the development of any new lesion

Brain MRI was done 3 months later and demonstrated relative improvement of the prior ischemic lesions without any new lesion development (Fig. 5).

## Discussion and conclusions

Here we described a young patient for whom the diagnosis of BCNE was made considering the diagnostic work-up performed for him. BCNE is a difficult diagnosis and relatively uncommon condition. Thrombotic endocarditis could be in the differential of the patient. However, since the patient was febrile and systolic murmur was detected on cardiac auscultation, IE is more likely. Currently empiric antibiotic treatment is recommended for BCNE [7]. Failure to culture the organism in BCNE may be due to the recent antibiotic use by the patient. Here, in this particular patient, parenteral and oral third-generation cephalosporin had been administered for his keratoconjunctivitis. This could be an explanation why the blood cultures were negative. Even though some uncommon pathogens may be the culprit in BCNE, more advanced laboratory tests performed for the presented patient at the tertiary referral center did not show any new organisms. Another possibility for BCNE which is less likely in the patient is due to systemic and connective-tissue diseases such as systemic lupus eryhtematosus [9]. Additionally, since the patient was not intravenous abuser or did not have any pre-existing chronic comorbidity the likelihood of fungal infections is low as supported by complementary laboratory tests performed later.

Transthoracic and transesophageal echocardiography can reveal vegetations in up to 90% of IE cases. Large, mobile vegetations on the valves are associated with an increased risk of brain embolization [10] as happened in the presented patient. For each millimeter increase in the size of vegetation, there is a 10% increase in the rate of ischemic lesions detected by brain MRI [11]. Ischemic stroke is the most common neurological complication of IE, affecting up to 35% of all patients and often it occurs due to embolism [12]. The neurologic abnormalities of the current patient also resolved gradually after heparin therapy. Heparin is used in the treatment and prevention of some major conditions such as pulmonary embolism [13] and coronary heart disease [14]. Heparin has been shown to significantly accelerate thrombin inhibition, which is subsequently inhibited by antithrombin co-factors, including Xa, IXa, XIa and XIIa, which are major contributors to the clotting cycle in our body [15].

The role of anticoagulant therapy in IE is controversial. Some guidelines discourage use of anticoagulation therapy in IE [12]. However, there is limited evidence in the literature regarding the role of anticoagulation treatment in IE. For instance, in a young patient with IE (blood culture positive for *Streptococcus mutans*) and embolic stroke, low molecular weight heparin was started in addition to antibiotics and the patient showed dramatic improvement in muscle force and dysarthria [16]. Unfortunately no clinical trials, as the best if our knowledge, exist regarding use of anticoagulation therapy in IE. Kitano et al. presented a 51-year-old female patient with lupus who had Libman-Sacks endocarditis [17]. The patient was treated with apixaban apart from the treatment of his underlying disease, but it was switched to heparin because it had no effect on the clearance of aortic valve vegetation. TTE after 1 week revealed dramatic but incomplete fading of the vegetation. They reported that although vegetations on the aortic valve grew while the patient was receiving apixaban, they diminished in size after starting heparin.

Jennings et al. reported an 81-year-old patient with a history of aortic valve replacement was presented with slurred speech [18]. Despite negative blood cultures, biopsy revealed *Staphylococcus epidermidis* after aortic root replacement due to endocarditis. In her treatment protocol, the patient was initially given thrombolysis, but after 2 h, extensive symptoms of brain injury developed and CT scan showed cerebral vascular obstruction. In other words, as the vegetations were separated from the aortic valve, the clots were trapped in the cerebral vessels. Finally, the authors concluded that thrombolysis was not successful generally and may be associated with an increased risk of stroke in the patient [18]. Over a 6-year period, Davis et al. [19] studied more than 250 patients with IE. Sixteen patients (6.2%) had negative blood cultures and 50 patients received adjuvant treatment with heparin. They concluded that the use of anti-coagulants did not appear to affect stroke, cerebral hemorrhage or mortality in patients with left-sided IE. Continuation of anticoagulant in patients with left-sided endocarditis should be considered in the absence of other contraindications. As such, the use of anti-coagulants in left-sided IE remains controversial.

BCNE, although uncommon, could be considered in patients with persistent fever and abnormal neurologic findings. This diagnosis becomes more likely when systemic antibiotics have been administered for patients. TTE should be performed to evaluate cardiac valves. Empiric antibiotics should be started immediately. In addition to antibiotics, when several vegetations are seen on cardiac valves, especially large vegetations and both the ventricular wall and the mitral valve, intravenous heparin, if there is no contraindication, might be helpful in management of individual patients considering all aspects of benefits and hazards of anticoagulation. In our experience, we observed a good response when intravenous heparin was administered for the patient. Both valvular vegetations and neurologic abnormalities resolved, although abnormal gait and slurred speech took longer to resolve completely.

## Data Availability

The necessary clinical data of the patient are presented in the case report. There is no dataset to be shared in public repositories.

## Abbreviations

BCNE: Blood culture-negative endocarditis
ED: Emergency department
ICU: Intensive care unit
IE: Infective endocarditis
MV: Mitral valve
RV: Right ventricle
TTE: Transthoracic echocardiography
TV: Tricuspid valve
WBC: White blood cell
HIV: Human immune deficiency
HTLV: Human T-lymphotropic virus
ANA: Antinuclear antibody
